# Adaptive dosing of immunotherapy plus anti-angiogenic therapy in a nonagenarian with hepatocellular carcinoma: a case report with 10-year survival

**DOI:** 10.3389/fonc.2026.1833336

**Published:** 2026-08-05

**Authors:** Pengfei Cui, Guannan Sun, Xueguang Guo, Jing Yang, Nijia Chang, Haili Huang, Na Li, Yi Hu, Fan Yin, Dong Zhang

**Affiliations:** 1 Department of Oncology, The Second Medical Center of Chinese People's Liberation Army (PLA) General Hospital, Beijing, China; 2 Department of Neurosurgery, The 983rd Hospital of the People’s Liberation Army, Tianjin, China; 3 Senior Department of Oncology, The Fifth Medical Center of PLA General Hospital, Beijing, China; 4 Department of Geriatrics, The Second Medical Center of Chinese PLA General Hospital, Beijing, China

**Keywords:** case report, geriatric oncology, hepatocellular carcinoma, immunotherapy, nonagenarian

## Abstract

**Background:**

Elderly cancer patients, particularly those aged ≥90 years, are significantly underrepresented in clinical trials, leaving a critical evidence gap for this growing population.

**Case presentation:**

We report a 90-year-old man with multiple comorbidities (coronary artery disease, hypertension, type 2 diabetes, hyperlipidaemia, hepatic steatosis, and hypothyroidism) and an ECOG performance status 2, who was diagnosed with hepatocellular carcinoma (HCC) in 2015. After initial microwave ablation in 2017 and for recurrence in 2021, he developed multifocal intrahepatic progression in January 2023 at age 97. Following progression on pembrolizumab monotherapy (100 mg q3w) and intolerance to lenvatinib combination, treatment was adapted to reduced-dose pembrolizumab (100 mg q3w) plus reduced-dose bevacizumab (3.75 mg/kg q3w)—approximately half and one-quarter of standard doses, respectively. He achieved partial response after three cycles with significant symptomatic improvement. As of March 2025 (age 99), he has completed 23 cycles with a progression-free survival of 15 months. He maintains functional independence, satisfactory symptom control (liver pain NRS 1–2), and only grade 1 fatigue, with no treatment-related hospitalizations.

**Conclusion:**

To our knowledge, this is the oldest reported patient with HCC to receive combined immunotherapy and anti-angiogenic therapy. This case suggests that with carefully adapted dosing—using approximately half-dose pembrolizumab and quarter-dose bevacizumab—a nonagenarian with multiple comorbidities may achieve prolonged disease control, challenging the conventional ‘one-size-fits-all’ dosing paradigm and highlighting the feasibility of individualized treatment strategies in the oldest-old population with HCC.

## Introduction

The global population is aging at an unprecedented pace, yet older adults—particularly those aged 80 years and above—remain profoundly underrepresented in cancer clinical trials (1). This persistent exclusion creates a critical evidence gap, leaving clinicians with little guidance when managing cancer in the oldest-old population, especially when patients present with multiple comorbidities and reduced performance status (1).

Hepatocellular carcinoma (HCC) is increasingly diagnosed in older individuals, driven by the demographic aging and improved surveillance programs. In recent years, the combination of immune checkpoint inhibitors and anti-angiogenic agents has emerged as the standard first-line treatment for advanced HCC, demonstrating significant survival benefits in pivotal phase III trials (2, 3). However, these landmark studies, including the IMbrave150 trial and subsequent real-world cohorts, have systematically excluded patients aged ≥90 years (2, 4). Consequently, no evidence-based recommendations exist for managing HCC in nonagenarians, and the risk-benefit profile of modern combination therapies in this population remains entirely unknown (5). At the time systemic therapy was initiated, the patient maintained Child-Pugh class A liver function (score 5) and an ECOG performance status of 2. The tumour was staged as BCLC stage C (multifocal intrahepatic disease, ECOG PS 2, no extrahepatic metastasis) and CNLC stage IIIa.

Herein, we present a unique case of a nonagenarian patient with HCC who, through an adaptive and carefully tailored therapeutic approach, achieved sustained overall survival exceeding 10 years from initial diagnosis—far surpassing the zero survival threshold predicted by his Charlson Comorbidity Index (CCI)—while maintaining excellent quality of life. To our knowledge, based on a systematic search of PubMed, Web of Science, and CNKI databases using the keywords ‘hepatocellular carcinoma’, ‘nonagenarian’, ‘immunotherapy’, ‘bevacizumab’, and ‘pembrolizumab’, this represents the oldest reported patient with HCC to receive combined immunotherapy and anti-angiogenic therapy. This case provides clinically relevant insights into the feasibility, dosing strategies, and outcomes of modern cancer therapy in the oldest-old population, highlighting the urgent need for inclusive research methodologies in geriatric oncology.

## Patient characteristics

A 90-year-old man presented in 2015 with a solitary liver lesion detected on routine health examination. His medical history was significant for multiple comorbidities, including coronary artery disease, hypertension, type 2 diabetes mellitus, hyperlipidaemia, hepatic steatosis, and hypothyroidism (managed with levothyroxine replacement). At the time of systemic therapy initiation in January 2023 (age 97), his Eastern Cooperative Oncology Group (ECOG) performance status was 2 and liver function was Child-Pugh class A (score 5). Detailed baseline characteristics are summarized in **Table 1**.

**Table 1:** Patient characteristics at baseline and key time points.

Characteristic	Value
Demographics	
Age at initial diagnosis (2015)	90 years
Age at start of systemic therapy (2023)	97 years
Age at last follow-up (March 2025)	99 years
Sex	Male
ECOG performance status (at systemic therapy start)	2
Comorbidities	
Cardiovascular	Coronary artery disease, hypertension
Endocrine	Type 2 diabetes mellitus, hyperlipidaemia, hypothyroidism (on levothyroxine)
Hepatic	Hepatic steatosis
Liver function at systemic therapy initiation (January 2023)	
Child-Pugh class	A (score 5)
Albumin	38.5 g/L
Bilirubin	15.2 μmol/L
ALT/AST	34/41 U/L
Ascites	None
Laboratory findings at systemic therapy initiation (January 2023)	
Renal function	Cr 82 μmol/L, eGFR 74 mL/min/1.73 m²
Complete blood count	Hb 118 g/L, WBC 4.2×10^9^/L, PLT 156×10^9^/L
Coagulation	PT 13.1 s, INR 1.08
Tumour characteristics	
Initial presentation (2015)	1.4 cm solitary lesion, segment S8
Histology (2017 biopsy)	Moderately to poorly differentiated HCC (Edmondson grade II–III); IHC: HepPar-1 (+), GPC-3 (+), CK19 (−), Ki-67 (approximately 15%)
AFP levels	2015 (initial diagnosis): 12.3 ng/mL (normal <20 ng/mL); 2017 (pre-MWA): 87.6 ng/mL; post-MWA 2017: normalised; January 2023 (systemic therapy start): 1,246 ng/mL; January 2024 (after 3 cycles): 186 ng/mL; January 2025: 94 ng/mL
Disease status at systemic therapy start (2023)	Multifocal intrahepatic progression, no extrahepatic metastasis; BCLC stage C, CNLC stage IIIa
Prior local therapies	
2017	Ultrasound-guided microwave ablation (MWA)
2021	Repeat MWA for local recurrence
Systemic treatment history	
First-line (Jan–Apr 2023)	Pembrolizumab 100 mg q3w (4 cycles) → progressive disease
Second-line (Apr–Oct 2023)	Pembrolizumab 100 mg q3w + lenvatinib (8 mg → 4 mg qod) → intolerance and progressive disease
Third-line (Oct 2023 – present)	Pembrolizumab 100 mg q3w + bevacizumab 3.75 mg/kg q3w → partial response, ongoing
Best response to current regimen	Partial response (RECIST 1.1)
Progression-free survival (current regimen)	15+ months (as of March 2025)
Quality of life and tolerability	
Adverse events on current regimen	Mild fatigue only (grade 1), no grade 3–4 events
Current symptoms	Mild fatigue; otherwise asymptomatic, independent living
Adverse event monitoring	CTCAE v5.0 assessed before each cycle, including clinical evaluation and laboratory tests (complete blood count, liver/renal/thyroid function, urinalysis)

ECOG, Eastern Cooperative Oncology Group; HCC, hepatocellular carcinoma; IHC, immunohistochemistry; MWA, microwave ablation; q3w, every 3 weeks; qod, every other day; RECIST, Response Evaluation Criteria in Solid Tumours; CTCAE, Common Terminology Criteria for Adverse Events; AFP, alpha-fetoprotein; ALT, alanine aminotransferase; AST, aspartate aminotransferase; Cr, creatinine; eGFR, estimated glomerular filtration rate; Hb, haemoglobin; WBC, white blood cell count; PLT, platelet count; PT, prothrombin time; INR, international normalised ratio.

## Initial presentation and local therapy (2015-2021)

In 2015, abdominal computed tomography (CT) revealed a 1.4 cm lesion in segment S8 of the right hepatic lobe, suggestive of primary liver malignancy. Given the patient’s advanced age, comorbidity burden, and small lesion size, he and his physicians elected for active surveillance with serial imaging. A structured surveillance protocol was implemented, with ultrasound and alpha-fetoprotein (AFP) monitoring every 3–6 months. This conservative approach was preferred over immediate MRI or invasive biopsy at that time, considering the patient’s age, the limited life expectancy associated with his Charlson Comorbidity Index, and the potential risks of procedural complications in a frail older adult. Serial imaging demonstrated stability of the lesion until 2017, when routine ultrasound follow-up revealed interval growth, prompting MRI evaluation and subsequent microwave ablation.

In 2017, follow-up magnetic resonance imaging (MRI) demonstrated interval enlargement of the lesion to 2.8 cm, with imaging features consistent with hepatocellular carcinoma (HCC). The patient underwent ultrasound-guided microwave ablation (MWA). Intraoperative biopsy specimens confirmed the diagnosis of moderately to poorly differentiated HCC. Post-ablation imaging showed a complete response with no residual enhancement.

In 2021, routine MRI identified a recurrent 2.2 cm nodule adjacent to the prior ablation zone, without evidence of additional lesions or extrahepatic spread. The patient underwent a second MWA procedure, achieving complete radiological ablation.

## Systemic therapy for advanced disease (2023-present)

The decision to initiate systemic therapy in January 2023 was preceded by a multidisciplinary discussion involving oncologists, geriatricians, and the patient and his family, focusing on goals of care. Several therapeutic options were considered: (1) best supportive care, which was deemed insufficient given the patient’s preserved functional status and willingness to pursue active treatment; (2) sorafenib or lenvatinib monotherapy, which were considered but deferred due to concerns about their broad toxicity profiles in a frail nonagenarian; and (3) immunotherapy-based regimens. Pembrolizumab was chosen as the initial agent based on its established efficacy in Asian patients with advanced HCC from the KEYNOTE-394 trial and its formulary availability (6). Although atezolizumab-bevacizumab was the established standard first-line combination at that time, we deliberately deferred upfront dual-agent therapy and adopted a step-up strategy (monotherapy first) given the patient’s age, ECOG PS of 2, and the unknown tolerability of combination therapy in this specific demographic.

At the time of systemic therapy initiation in January 2023, the patient’s alpha-fetoprotein (AFP) level was 1,246 ng/mL (normal <20 ng/mL), a substantial increase from 87.6 ng/mL before the first microwave ablation in 2017, after which AFP had normalised. Laboratory evaluation revealed preserved liver function (Child-Pugh class A, score 5) with albumin 38.5 g/L, total bilirubin 15.2 μmol/L, and ALT/AST 34/41 U/L. Renal function was adequate (creatinine 82 μmol/L, eGFR 74 mL/min/1.73 m²), and haematological parameters were within normal limits (haemoglobin 118 g/L, white blood cell count 4.2×10^9^/L, platelet count 156×10^9^/L). Coagulation function was preserved (prothrombin time 13.1 seconds, INR 1.08).

In January 2023, at the age of 97, follow-up imaging revealed multifocal intrahepatic progression involving multiple segments, without extrahepatic metastasis. Based on these findings, the patient was staged as BCLC C (multifocal intrahepatic disease, ECOG PS 2) and CNLC IIIa. Liver function remained Child-Pugh class A (score 5). After comprehensive discussion with the patient and his family regarding goals of care, the decision was made to initiate systemic therapy. After comprehensive discussion with the patient and his family regarding goals of care, the decision was made to initiate systemic therapy.

### First-line immunotherapy

Given the patient’s advanced age (97 years), ECOG performance status 2, and multiple comorbidities, we adopted the geriatric pharmacology principle of “start low, go slow” and initiated reduced-dose pembrolizumab at 100 mg intravenously every 3 weeks—approximately 50% of the standard dose of 200 mg used in pivotal trials (7). After four cycles, restaging MRI according to RECIST 1.1 criteria confirmed progressive disease (PD), with enlargement of existing lesions and appearance of new intrahepatic lesions.

### Combination with lenvatinib

The multi-kinase inhibitor lenvatinib was added at a reduced dose of 8 mg daily (the standard dose in clinical trials is 12 mg daily for patients with body weight ≥60 kg (7)). However, the patient gradually developed a series of adverse reactions, including moderate fatigue (grade 2), anorexia (grade 2), nausea (grade 1), and hoarseness (grade 2), leading to inconsistent oral adherence. Dose reduction to 4 mg every other day improved tolerability but failed to halt disease progression, evidenced by worsening right upper quadrant pain [Numerical Rating Scale (NRS) score increasing from 2–3 to 6-7] and radiographic tumour growth on follow-up MRI.

### Adaptation to bevacizumab combination

In October 2023, after multidisciplinary discussion, therapy was adjusted to pembrolizumab (100 mg q3w) combined with bevacizumab at a substantially reduced dose of 3.75 mg/kg intravenously every 3 weeks. This represents approximately half the standard pembrolizumab dose and one-quarter of the standard bevacizumab dose used in phase III trials (where bevacizumab is typically administered at 15 mg/kg q3w) (2, 3). The rationale for selecting bevacizumab over other TKIs was based on its selective VEGF inhibition, which offers a more favourable tolerability profile in frail older adults, and the vascular normalization hypothesis, whereby lower anti-angiogenic doses may suffice to remodel the immunosuppressive tumour microenvironment and enhance immunotherapy efficacy (8).

### Adverse event monitoring

Throughout the treatment course, adverse events were systematically assessed and graded at each cycle prior to drug administration according to the Common Terminology Criteria for Adverse Events (CTCAE) v5.0 (9), based on clinical evaluation and laboratory tests including complete blood count, renal and liver function tests, thyroid function tests, and urinalysis. Grade ≥ 3 adverse events were defined as the primary safety endpoint for treatment modification or discontinuation.

## Outcomes

**Figure 1:**
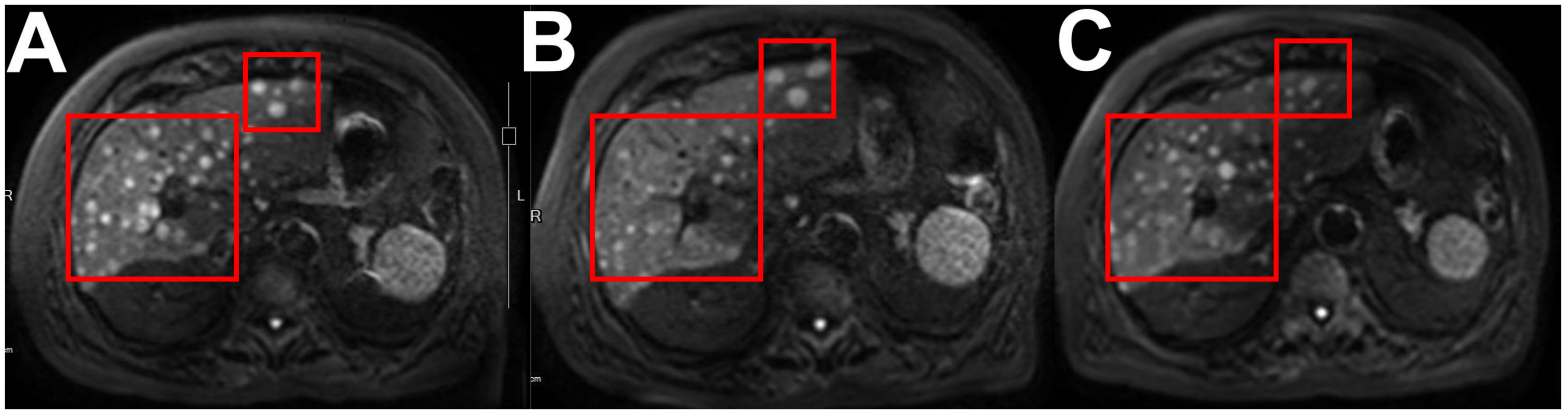
MRI images showing tumour response to pembrolizumab plus bevacizumab. Axial contrast-enhanced T2-weighted MRI images at three time points during treatment with reduced-dose pembrolizumab plus bevacizumab. Due to the diffuse, multifocal nature of the intrahepatic disease—with numerous small nodules distributed across both lobes—representative images are shown rather than individual measured target lesions, as the latter would not have been reliably selectable or reproducible per RECIST 1.1 criteria. The lesions appear as hyperintense foci (white) on T2-weighted imaging, corresponding to areas of viable tumour, which were confirmed by arterial phase hyperenhancement on dynamic contrast-enhanced sequences. **(A)** October 2023 (pre-treatment): Baseline imaging before initiation of pembrolizumab-bevacizumab, showing multifocal intrahepatic progression with hyperintense lesions within the red boxes. **(B)** January 2024 (after 3 cycles): Follow-up imaging after three cycles of combination therapy, demonstrating partial response (PR) according to RECIST 1.1 criteria, with significant reduction in size and signal intensity of the lesions. **(C)** January 2025 (15 months): Imaging after 15 months of continuous treatment, showing sustained response with no evidence of new lesions or progression.

**Figure 2:**
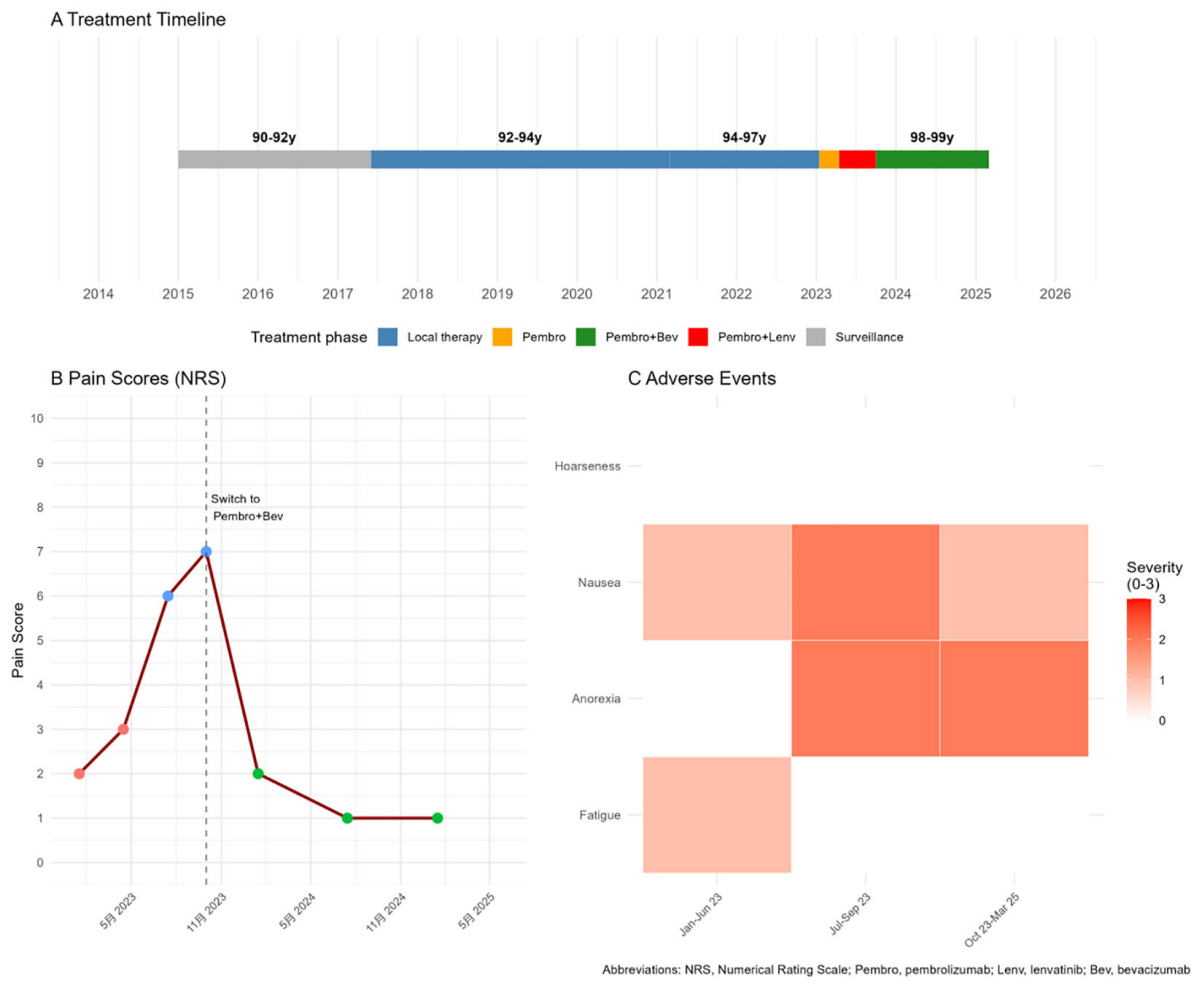
Clinical course of combined immunotherapy and anti-angiogenic therapy in a nonagenarian patient with advanced hepatocellular carcinoma. **(A)** Treatment timeline. Coloured horizontal bars represent different treatment phases from initial diagnosis in 2015 to the most recent follow-up in March 2025. All bars are aligned on a single horizontal axis to emphasize temporal continuity. Phase colours: Surveillance (grey), local therapy with microwave ablation (steel blue), pembrolizumab monotherapy (orange), pembrolizumab plus lenvatinib (red), and pembrolizumab plus bevacizumab (forest green). Age ranges (in years) are displayed above selected bars (Surveillance: 90-92y; first local therapy: 92-94y; second local therapy: 94-97y; pembrolizumab-bevacizumab: 98-99y). Age labels are omitted for the short-duration pembrolizumab and pembrolizumab-lenvatinib phases to avoid overlap. **(B)** Pain scores during advanced disease phase. Pain intensity measured by the Numerical Rating Scale (NRS, range 0–10) from January 2023 to January 2025. Higher scores indicate more severe pain. Points are coloured by treatment regimen: pembrolizumab monotherapy (orange), pembrolizumab plus lenvatinib (red), and pembrolizumab plus bevacizumab (green). The vertical dashed line marks the switch from pembrolizumab-lenvatinib to pembrolizumab-bevacizumab in October 2023. A sharp decline in pain scores is observed following regimen adjustment. **(C)** Adverse event profile. Heatmap showing severity of treatment-related adverse events across three time periods corresponding to different treatment phases: January–June 2023 (pembrolizumab monotherapy), July–September 2023 (pembrolizumab plus lenvatinib), and October 2023 – March 2025 (pembrolizumab plus bevacizumab). Symptom severity is graded according to CTCAE criteria (0 = none, 1 = mild, 2 = moderate, 3 = severe) and represented by colour intensity (white to red). The pembrolizumab-bevacizumab phase shows marked reduction in adverse events, with only mild fatigue persisting. NRS, Numerical Rating Scale; Pembro, pembrolizumab; Lenv, lenvatinib; Bev, bevacizumab; CTCAE, Common Terminology Criteria for Adverse Events.

Remarkably, after three cycles of the adapted regimen, restaging contrast-enhanced MRI demonstrated a partial response (PR) according to RECIST 1.1 criteria. Due to the multifocal, diffusely distributed nature of the intrahepatic lesions—with multiple small nodules scattered across both hepatic lobes and some lesions lacking clearly defined margins—quantitative measurement of individual target lesions per RECIST 1.1 was not feasible. Instead, the overall tumour burden was assessed based on the following integrated criteria: (1) a marked reduction in the number and size of all measurable lesions; (2) a significant decrease in arterial phase hyperenhancement on contrast-enhanced MRI, indicating reduced viable tumour activity; (3) the absence of new lesions; and (4) a parallel decline in serum alpha-fetoprotein (AFP) from 1,246 ng/mL at baseline to 186 ng/mL after three cycles (an 85% reduction), which served as a corroborative quantitative biomarker of tumour response. Collectively, these findings support the assessment of partial response (**Figure 1A**). This radiological response was accompanied by dramatic symptomatic improvement, with liver area pain decreasing from NRS 6–7 to 1–2 (**Figure 2B**).

As of March 2025, at the age of 99, the patient has completed 23 cycles of pembrolizumab-bevacizumab combination therapy, achieving progression-free survival (PFS) of 15 months from initiation of this regimen. Sustained radiological response was confirmed at the 15-month follow-up (**Figure 1B**). He continues to receive treatment with excellent tolerability, reporting only mild fatigue (grade 1) with no grade 3–4 adverse events observed (**Figure 2C**). His most recent laboratory evaluation shows stable liver function (Child-Pugh class A, score 5) and normal thyroid function on levothyroxine replacement. He remains living independently, with preserved functional status and satisfactory symptom control (liver pain decreased from NRS 6–7 to 1–2), reporting only grade 1 fatigue with no grade ≥3 adverse events, and no hospitalizations related to treatment or disease progression. The clinical course, pain scores, and adverse event profile are summarized in **Figure 2**.

## Discussion

This case presents several unique and clinically significant aspects that merit careful consideration in the context of an aging global population and the paucity of evidence guiding cancer treatment in the oldest-old. More importantly, the dosing strategy employed in this patient warrants detailed examination, as it may provide a practical reference for managing similar frail older adults.

The clinical decision-making process in this case was guided by a patient-centred framework that prioritized safety, functional preservation, and iterative re-assessment. For the initial surveillance period, active monitoring with regular ultrasound and AFP was chosen over immediate invasive procedures, reflecting a careful risk–benefit balance in a 90-year-old with significant comorbidities. When systemic therapy became necessary, we considered multiple alternatives—including best supportive care, TKI monotherapy, and combination immunotherapy—and ultimately selected a step-up immunotherapeutic strategy based on the patient’s treatment goals, performance status, and the principle of minimizing initial toxicity while preserving the option for treatment intensification. The subsequent switch from lenvatinib to bevacizumab, rather than to other TKIs such as regorafenib or cabozantinib, was driven by bevacizumab’s favourable tolerability profile as a selective VEGF inhibitor and the emerging evidence supporting reduced-dose anti-angiogenic therapy in combination with immunotherapy. This sequential, response-adapted approach—enabled by close multidisciplinary monitoring—allowed us to tailor therapy to the patient’s evolving clinical status while maintaining treatment efficacy.

First, to our knowledge, this represents the oldest reported patient with hepatocellular carcinoma (HCC) receiving combined immunotherapy and anti-angiogenic therapy. We systematically searched PubMed, Web of Science, and CNKI databases (up to March 2025) using the keywords ‘hepatocellular carcinoma’, ‘nonagenarian’, ‘immunotherapy’, ‘bevacizumab’, ‘pembrolizumab’, and their combinations. No published case reports of HCC patients aged ≥90 years receiving combined immunotherapy and anti-angiogenic therapy were identified. While subgroup analyses of phase III trials have included older adults—for example, the IMbrave150 trial reported outcomes in patients aged ≥65 years (2)—and real-world cohorts have extended observations to octogenarians (4), none have enrolled or described patients aged ≥90 years. This case demonstrates that chronological age alone should not serve as an absolute contraindication to modern combination therapies, provided careful attention is paid to individual patient characteristics, comorbidity burden, and functional status.

Second, the adaptive dosing strategy employed here achieved a remarkable balance between efficacy and tolerability. The patient received approximately 50% of the standard pembrolizumab dose (100 mg vs. 200 mg q3w) and 25% of the standard bevacizumab dose (3.75 mg/kg vs. 15 mg/kg q3w) used in pivotal registration trials (2, 7). The initial dose selection was guided by the geriatric pharmacology principle of ‘start low, go slow’ (10), given the patient’s advanced age (97 years), ECOG performance status of 2, and multiple comorbidities. The decision to reduce the starting doses was informed by multiple converging clinical factors: (1) the patient’s ECOG performance status of 2, indicating reduced functional capacity; (2) multiple comorbidities including coronary artery disease, hypertension, type 2 diabetes, and hepatic steatosis, which increase the risk of treatment-related complications; (3) polypharmacy (including levothyroxine, antihypertensives, and oral hypoglycaemics), raising concerns about drug-drug interactions and adherence; and (4) chronological age of 97 years, associated with age-related physiological changes affecting drug pharmacokinetics and pharmacodynamics. Collectively, these factors—rather than a single variable—guided the empirical dose selection. However, it is important to recognise that, unlike cytotoxic chemotherapy, immune-related adverse events (irAEs) are not consistently dose-dependent (11), and dose reduction of immune checkpoint inhibitors does not necessarily translate into improved safety (5). The empiric dose reduction to 100 mg was therefore based on clinical judgment and frailty assessment rather than on a well-established pharmacokinetic or pharmacodynamic rationale for immunotherapy. Indeed, pembrolizumab at this reduced dose was associated with disease progression when used as monotherapy, suggesting that the reduced dose alone was insufficient to achieve tumour control. The subsequent response likely resulted from the synergistic effect of bevacizumab-induced vascular normalisation—which may enhance immune cell infiltration and T-cell activity—rather than from the reduced pembrolizumab dose per se. This underscores the need for further research to define the optimal biological dose of immunotherapy in frail older adults, as well as predictive biomarkers of response and toxicity in this population. Notably, pivotal phase III trials, including IMbrave150, systematically excluded patients aged ≥90 years, leaving the safety profile of standard doses entirely unknown in this population (2). Thus, our empirical dose reduction was a deliberate risk–benefit decision based on comprehensive geriatric assessment rather than pharmacokinetic data. Despite these substantial dose reductions—implemented empirically based on geriatric assessment and clinical judgment—the patient achieved partial response and prolonged progression-free survival (PFS) exceeding 15 months. The radiological response was paralleled by a marked decline in AFP levels, from 1,246 ng/mL at baseline to 186 ng/mL after three cycles, and further to 94 ng/mL at 15 months, corroborating the objective tumour response. This notably exceeds the median PFS reported in the KEYNOTE-394 trial of pembrolizumab monotherapy (approximately 2.6 months) (6, 7) and compares favourably with the PFS observed with standard-dose atezolizumab-bevacizumab combinations (approximately 6–8 months) (2). This observation suggests the potential value of exploring individualized, response-adapted dosing strategies in frail older adults and may encourage further investigation into such approaches. The concept of “start low, go slow”—well-established in geriatric pharmacology—may have meaningful application in oncology for the oldest-old population.

Third, this case illustrates the critical importance of therapeutic flexibility and close monitoring. When the initial combination with lenvatinib proved both intolerable (despite dose reduction) and ineffective, the timely switch to bevacizumab—a mechanistically distinct anti-angiogenic agent targeting the VEGF pathway rather than multiple kinases—was associated with treatment response. The rationale for selecting bevacizumab over other TKIs was twofold. First, lenvatinib’s broad multi-kinase inhibition (targeting VEGFR1-3, FGFR1-4, RET, and KIT) contributed to its poor tolerability profile in this frail patient, whereas bevacizumab, as a selective VEGF monoclonal antibody, offers theoretically better tolerability. Second, the ‘vascular normalisation’ hypothesis suggests that lower doses of anti-angiogenic agents may suffice to remodel the immunosuppressive tumour microenvironment and enhance immunotherapy efficacy (8). This is supported by recent real-world evidence showing that reduced-dose bevacizumab does not compromise survival outcomes—a cohort study of 354 patients receiving first-line combination immunotherapy for advanced HCC reported no significant differences in PFS (11.2 vs 14.8 months, p=0.5) or OS (20.4 vs 26.8 months, p=0.1) between reduced-dose and full-dose groups (12). We also acknowledge that the dose selection for bevacizumab was empiric and not guided by formal pharmacokinetic or pharmacodynamic data. The optimal biological dose of bevacizumab for vascular normalisation may be lower than the maximum tolerated dose, but the precise threshold remains undefined in the oldest-old population. Furthermore, several clinically relevant bevacizumab-related toxicities—such as thromboembolic events, gastrointestinal perforation, and major bleeding—are not clearly dose-dependent, and their occurrence is more closely related to patient-specific factors (e.g., age, cardiovascular status, tumour location, and antithrombotic use). In this patient, the absence of these toxicities may reflect careful patient selection and close monitoring rather than dose reduction alone. Thus, our dosing strategy should be viewed as hypothesis-generating rather than evidence-based, and requires validation in larger prospective studies. This “adaptive therapy” approach, guided by real-time assessment of both tumour response and patient tolerance, appears particularly well-suited to the geriatric oncology population, where treatment goals often prioritize quality of life and functional independence alongside longevity.

Several alternative explanations for the observed tumour response should be considered. First, the delayed onset of immunotherapy effects is well recognised, and it is possible that the partial response observed after three cycles of pembrolizumab-bevacizumab reflected a late response to pembrolizumab monotherapy that had not yet manifested at the time of progression assessment after four cycles. Second, although MRI assessment was performed according to RECIST 1.1 criteria and reviewed by experienced radiologists, some variability in imaging interpretation cannot be entirely excluded. Third, bevacizumab, independent of immunotherapy, has intrinsic anti-tumour activity in HCC via VEGF pathway inhibition, and may have contributed to the observed response. Thus, the response was likely multifactorial and cannot be attributed exclusively to the combination or to either agent alone.

Fourth, the patient’s survival trajectory defies conventional prognostic estimates. Based on his age (90 years at diagnosis) and Charlson Comorbidity Index (CCI) score (reflecting multiple comorbidities), predicted 10-year survival would approach zero. Yet this patient not only surpassed that threshold but maintained good functional status and symptom control throughout—remaining independent, free from hospitalization, with liver area pain well controlled (NRS 1–2) and only grade 1 fatigue on his most recent assessment. This outcome illustrates the potential of appropriately tailored cancer therapy, even in the oldest-old, and highlights the limitations of conventional prognostic tools when applied to this population.

The management of cancer in nonagenarians requires a paradigm shift from disease-centred to patient-cantered care. For this population, traditional endpoints such as overall survival must be balanced against functional outcomes, symptom control, and preservation of independence. Our approach—comprehensive geriatric assessment, shared decision-making, low-dose initiation, and continuous adaptation based on tolerance—may serve as a template for managing similar patients. The involvement of multidisciplinary teams, including geriatricians, oncologists, and palliative care specialists, is essential to navigate the complex trade-offs inherent in treating advanced cancer in frail older adults.

### Limitations

As a single case report, these findings are hypothesis-generating and not generalizable. The optimal dosing strategy for nonagenarians remains undefined, and the favourable outcomes observed in this patient should not be interpreted as evidence of efficacy or safety applicable to all nonagenarian patients with HCC. The dose reductions implemented were empirical rather than based on pharmacokinetic data, and our approach required intensive monitoring and frequent adjustments that may not be feasible in all clinical settings. Additionally, quality of life was assessed clinically based on functional status, symptom control, and adverse event profiles rather than with validated instruments such as the EORTC QLQ-C30 or HCC-specific scales. This limits the objective quantification of the patient’s quality of life and functional outcomes. Finally, the absence of geriatric assessment scores (e.g., G8 or VES-13) limits objective characterization of frailty. Furthermore, the diffuse distribution of intrahepatic lesions precluded formal target lesion selection and measurement according to RECIST 1.1, necessitating a qualitative and semiquantitative assessment of overall tumour burden based on the reduction in lesion number, loss of arterial phase enhancement, and absence of new lesions. While AFP decline provided an objective quantitative correlate, the absence of standardised target lesion measurements limits the precision of the radiological response assessment.

### Implications for research and practice

This case highlights the urgent need for inclusive clinical trial designs that accommodate the oldest-old population. Pragmatic trials, registry-based studies, and innovative methodologies such as n-of-1 trials or Bayesian adaptive designs may help generate evidence for this underserved population. Future research should focus on defining optimal dosing strategies, identifying predictive biomarkers of efficacy and toxicity in older adults, and developing validated tools for real-time assessment of treatment tolerance (13, 14). In the meantime, clinicians must rely on careful individualization of therapy, guided by principles of geriatric assessment and adaptive treatment strategies, while engaging patients and families in shared decision-making about goals of care.

## Conclusion

This case suggests that with carefully adapted dosing and continuous monitoring, a nonagenarian patient with hepatocellular carcinoma and multiple comorbidities may achieve prolonged survival and maintained quality of life with combined immunotherapy and anti-angiogenic therapy. To our knowledge, this represents the oldest reported patient with HCC to receive such combination therapy, achieving 10-year overall survival from initial diagnosis and 15-month progression-free survival on the current regimen, with excellent tolerability.

The adaptive dosing strategy employed—utilizing approximately half-dose pembrolizumab and quarter-dose bevacizumab—challenges the conventional “one-size-fits-all” dosing paradigm and suggests that substantial dose reductions may preserve efficacy while ensuring tolerability in frail older adults. The successful switch from lenvatinib to bevacizumab following intolerance and progression further underscores the importance of therapeutic flexibility and real-time response assessment in this population.

As the global population ages, developing evidence-based approaches for treating cancer in the oldest-old becomes increasingly urgent. This case highlights the critical need for inclusive clinical trial designs that accommodate patients aged ≥90 years, as well as research into optimal dosing strategies, predictive biomarkers, and validated tools for assessing treatment tolerance. In the absence of such evidence, our experience suggests that flexible, patient-centred adaptive strategies—guided by comprehensive geriatric assessment, shared decision-making, and close monitoring—may offer a path forward, challenging the notion that age alone should limit access to effective cancer therapies.

## Data Availability

The original contributions presented in the study are included in the article/supplementary material. Further inquiries can be directed to the corresponding authors.
